# Targeting Translation Dependence in Cancer

**DOI:** 10.18632/oncotarget.218

**Published:** 2011-02-15

**Authors:** Abba Malina, Regina Cencic, Jerry Pelletier

**Affiliations:** ^1^ Department of Biochemistry, McIntyre Medical Sciences Building, McGill University, Montreal, Quebec, Canada; ^2^ The Rosalind and Morris Goodman Cancer Center, McIntyre Medical Sciences Building, McGill University, Montreal, Quebec, Canada

**Keywords:** Translation, Translational Control, eIF4F, eIF4E, eIF4A, Translation Inhibitor

## Abstract

A challenge in cancer therapy is to selectively target activities that are essential for survival of malignant cells while sparing normal cells. Translational control represents a potential anti-neoplastic target because it is exerted by major signaling pathways that are often usurped in cancers. Herein we describe approaches being developed that target eukaryotic initiation factor (eIF) 4F, a heterotrimeric complex that integrates multiple signaling inputs to the translation apparatus.

## INTRODUCTION

The single greatest challenge in the treatment of cancer has always been to uncover therapeutic agents that eliminate only tumor cells while sparing normal cells. The era of genomics has given a much deeper understanding of the biology of cancer and the genetic mutations that underlie progression of the disease and has brought with it a new generation of so-called molecular targeted cancer therapeutics that specifically act on the very oncogenic lesions that promote and sustain the disease. These new drugs rely on the fact that maintenance of transformed cells and the pathogenesis of the tumor are inextricably linked to the initial genetic reprogramming that has occurred, a concept referred to as “oncogene addiction” [1]. Nevertheless, most of the more common older-generation chemotherapeutics currently available for the oncologist have broad applicability for diverse neoplastic disease despite suffering from low therapeutic indices in patients. Thus, the notion of using “dirty” compounds for the treatment of cancer has yet to be supplanted in the clinic nor has it lost traction in current drug development, as can be seen in several recently FDA approved chemotherapeutics (e.g. bortezomib or sorafenib). In fact, what seems at first a contradiction to the whole notion of rational designed targeted-therapeutics can be explained in a model elegantly described by Elledge and colleagues as “non-oncogene addiction”[2]. This model puts forth the idea that the vast biological rewiring on which the tumorigenic state of the cell depends on stem from gene products that in and of themselves are not natural oncogenes and whose functions are now “rate-limiting” to the survival and proliferation of the transformed cell. It is this unhinged metabolic burden that renders cancer cells exquisitely dependant on intrinsic stress-relief pathways for their normal existence, offering up unique therapeutic opportunities. One such class of emerging cancer drug targets, protein synthesis inhibitors, will remain the focus of this minireview.

The mRNA translation process may be thought of as occurring in three phases: initiation, elongation, and termination. Initiation, as its name implies, is the process wherein the cell prepares mRNA transcripts for binding to ribosomal subunits and subsequent proper alignment of the ribosome to the correct initiation codon, thus allowing for proper polypeptide synthesis (and gene expression) to ensue. Elongation is polypeptide synthesis, the cycle of amino-acid covalent attachment into polypeptide chains catalyzed by the ribosome, the result of matching the triplet-nucleotide codons embedded in mRNA message to their cognate amino acid-acylated tRNA. Termination is the end stage of polypeptide synthesis, the halting of a transiting ribosome at a stop codon for which there are no corresponding amino acid-acylated tRNAs and thus release of the fully synthesized protein into the cell. Ribosome density measurements along mRNA templates are consistent with the notion that the initiation phase of translation is generally rate-limiting [3]. We refer the reader to other texts for more in-depth analysis of the processes of elongation and termination [4, 5], which are well beyond the purview of this review. What follows is a brief overview of the initiation process, as it elaborates on some of the factors targeted by the inhibitors discussed below (for a recent review and a more thorough analysis see Refs. [6, 7]).

Translation initiation is a multifaceted highly regulated biological process, requiring at least nine eukaryotic initiation factors (eIFs) composed from at least 30 subunits. It can be separated into three distinct steps: i) the binding of initiation factors to mRNA transcripts to prepare for, ii) the binding of the 43S ribosomal complex (which consists of the 40S ribosome subunit in complex with eIF2-GTP-tRNA-Met_i_ ternary complex, eIF3, eIF1, eIF1A and eIF5) to the mRNA message, thus forming the 48S initiation complex and the, iii) joining of the 40S ribosome to the 60S ribosomal subunit, once the 48S ribosome is properly aligned at the start codon of the open-reading frame. For the majority of mRNAs, the binding of 43S ribosomal complexes begins at the 5'-end, where the methyl-7-guanosine (or “cap”) structure is found. Here, a preformed 43S pre-initiation complex is directed to the 5'-end with the help of the eIF4F initiation complex. Once there the ribosome begins “scanning” the 5'-untranslated region (5'-UTR) unidirectionally towards the 3'-end, until it reaches the appropriate initiation codon. Once properly oriented, eIF5 and eIF5B, using the energy from the hydrolysis of two GTP molecules, help replace all the eIFs with the joining large 60S ribosomal subunit, forming an elongation-competent 80S ribosome. Although most mRNAs utilize the cap structure to facilitate recruitment of the 43S ribosomal complex, initiation on a select few cellular and viral mRNAs (typically those that bear no cap-structure or that have long and complex 5'-UTRs) is mediated by an alternative cap-independent mechanism, utilizing a distinct RNA structure termed an internal ribosome entry site (IRES), which directs binding and start codon selection of the 43S complex internally on the mRNA transcript [6, 8].

## EIF4F

**Figure 1:**
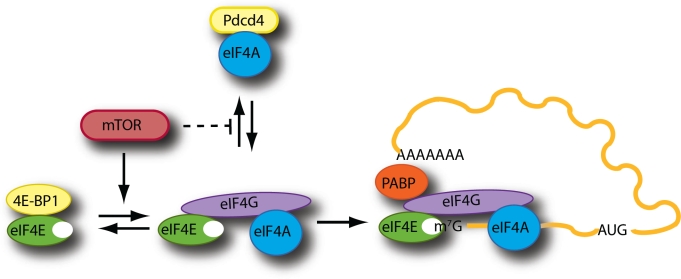
Schematic outline of mTOR regulation of the eIF4F-mRNA binding step

eIF4F is a heterotrimeric complex composed of: i) eIF4A, a DEAD-box containing ATPase and ATP-dependent RNA helicase required to melt local secondary structure and facilitate access of the ribosome to the mRNA template; ii) eIF4G, a modular scaffolding protein that mediates mRNA binding to the 43S pre-initiation complex, and iii) eIF4E, the cap-binding protein responsible for binding of the eIF4F complex to the mRNA cap structure [5, 9] (Fig. 1). In mammals, there are three isoforms of eIF4A (eIF4AI, II and III) that share ~90% and ~65% identity, respectively, with the most abundant cellular factor eIF4AI [10, 11]. All isoforms are DEAD-box RNA helicase family members but only the paralogs eIF4AI and eIF4AII are found in the eIF4F complex and participate in translation initiation [12, 13]. Similarly, there are two paralogs of eIF4G (eIF4GI and eIF4GII) that share 46% identity, as well as a distantly related protein, p97/DAP5/NAT1, that boasts a similar amino-acid sequence to the C-terminal portion of eIF4G. Despite being able to bind eIF4A and eIF3, p97 cannot bind eIF4E and thus appears to play a role in the cap-independent translation of a specific subset of mRNAs [14]. eIF4E imparts RNA binding specificity to the eIF4F complex by selectively recognizing the cap structure present at the 5' end of all eukaryotic cellular mRNAs [15-18]. Binding of 43S ribosomal complexes to the cap in order to begin scanning is thought to require eIF4F for most mRNAs. Unstructured mRNAs in *in vitro* reconstituted systems with purified translation components are able to weakly recruit 43S complexes [19], but the introduction of even a modest amount of RNA secondary structure eliminates all 43S recruitment in the absence of eIF4F. Once cap-bound, the various components of eIF4F act in concert to stabilize it to the mRNA template and prepare the mRNA for binding to 43S complexes. eIF4G, now proximally located on the mRNA template, reinforces the eIF4E-cap interaction [20-22]. eIF4A then unwinds local secondary structure to prepare the template for its interaction with 43S complexes, a process that is greatly improved by the auxiliary eIF4A interacting factors, eIF4B [23] and eIF4H [24]. It is eIF4G that directs 43S ribosome binding through a bridging interaction with eIF3, and finalizes 43S complex binding to the mRNA [25]. Although eIF4F primarily directs 5'-end 43S recruitment, it also brings the 3'-end in close proximity to the 5'-cap, through an interaction between eIF4G and the 3'-end bound poly(A) binding protein (PABP), enabling circularization of the mRNA template [26-28] (Fig. 1). This results in a stimulatory effect on ribosome binding and translation as a whole, either by ensuring that eIF4F remains cap-bound or through the recycling and reinitiation of ribosomes post-termination [27].

## EIF4F AND ONCOGENESIS - SEEING THE FOREST BUT NOT THE TREES

Ever since early experiments in model cell culture systems demonstrated that overexpression of eIF4E on its own can drive transformation of fibroblasts [29], there has emerged a large body of literature implicating eIF4F and several other translation initiation factors (e.g. eIF3 subunits, eIF2) in cancer biology [30]. The evidence supporting eIF4E as an oncogene is now quite compelling and has been demonstrated in multiple settings. In both xenograft nude-mouse and more genetically and clinically relevant mouse cancer models, overexpression of eIF4E was shown to both accelerate the onset of tumor formation and aggravate its drug response [29, 31, 32]. Conversely, overexpression of 4E-BP (for 4E-Binding Protein, a competitive inhibitor to the eIF4E-eIF4G interaction) in a p53^−/−^ mouse cancer model slowed progression of cancer in part through a mechanism involving premature senescence [33]. Moreover, in a wide variety of blood and solid tumor samples, elevated expression of eIF4E protein correlates with later stage tumors, more aggressive pathologies and poorer prognosis [34-49], while the opposite appears to be true for 4E-BP [50]. Similarly, eIF4GI overexpression appears to phenocopy some of the oncogenic features of eIF4E: it too can drive transformation of mouse cell lines [51] and increased expression has been found in some overlapping tumor tissues [52-54]. Finally, there have been a few reports which found eIF4A expression to be elevated in some human derived tumor samples, melanoma [55] and hepatocellular carcinoma [56] cells, although the implication of this is as of yet uncertain [57].

And yet, even with all of the aforementioned data demonstrating eIF4E (or more generally eIF4F) as a *bona fide* oncogene, the precise molecular mechanism governing its tumorigenicity has remained somewhat elusive. Although it might seem intuitive at first that cancer cells would benefit greatly from increased rates of protein of synthesis (and in fact they do), in the case of eIF4E this is not really the case, as the levels that elicit oncogenesis are somewhat modest (~2-3 fold) [31] and probably not high enough to alter overall protein abundance. More likely, the mechanism by which increased eIF4F activity contributes to transformation is via the increase in translation of a limited set of pro-oncogenic mRNA transcripts [58, 59]. One of the reasons for this pronounced selectivity is thought to be due to the differential requirements that some mRNAs have towards eIF4F activity [60]. mRNAs that have long, G-C rich and structured 5'-UTR nucleotide sequences, are generally poorly translated (presumably due to lowered 48S complex formation rates due to unfavorable eIF4F-cap interaction [61-63]), under most transient and normal growth conditions of the cell, where initiation factors in general, and eIF4F in particular, are limiting. In essence, they cannot outcompete the other more efficiently translated messages (those that have short and relatively unstructured 5'-UTRs) for ribosomes [60]. This all changes once the levels and activity of eIF4F rises: those messages that were once outcompeted will have their translation rates disproportionately stimulated, stemming from an increase in the rate-constant of ribosome binding and initiation due to the relative decrease in thermal stability at their 5'-ends mediated by increased eIF4F levels. Perhaps not surprisingly, a great many pro-growth, stress-associated or cell-cycle regulated transcripts whose protein levels are tightly controlled and are kept at a relatively low homeostatic level generally bear the hallmarks of a poorly translated message. In fact, simple overexpression of eIF4E can increase the levels of a wide variety of messages encoding growth and survival regulators (e.g. PDGF, FGF-2, VEGF), signal transducers (e.g. Pim-1, Ras), and components of the cell cycle and apoptotic machinery (e.g. cyclin D1, c-myc, RNR2, ODC, survivin and Mcl-1) [60, 64-68]. And this list is expanding given recent efforts at gene profiling of polysomal bound mRNAs that are under eIF4F regulation [69, 70]. Future technologies, such as those involving novel RNA-sequencing methodologies [3] will no doubt uncover an even more complex translational regulatory system. Thus targeting translation initiation, and in particular the eIF4F complex, would seem a viable and novel chemotherapeutic avenue.

## INHIBITING EIF4F – NOT ALL SUBUNITS ARE EQUAL

In recent years, there has been renewed interest in the development of pharmacological agents that disrupt these key steps of translation, in particular agents that block eIF4F activity. This has been based on a few early proof-of-principle experiments that showed that downregulation of eIF4E via antisense oligonucleotides [71, 72] or direct inhibition from peptides that compete for eIF4G binding [73] can stop transformation, cause cell-cycle arrest, and elicit apoptosis. But much of the attention paid of late to eIF4E biology as it pertains to cancer, especially from oncologists and pharmaceutical companies, relates to the anti-neoplastic potential of the macrolide rapamycin (and its analogs) and its cellular target, the master kinase mTOR. Note that a full treatment of the literature and published research on the subject of mTOR is well beyond the purview of this text and we encourage the reader to look at a recent review for a more complete understanding of the extensive and important role that mTOR plays in the cell [74]. Nevertheless, it is important to emphasize one key downstream target and effector of mTOR function, namely eIF4F (Fig 1).

mTOR is a master regulator of cellular homeostasis, sensing inputs from growth factor withdrawal, amino acid imbalance, energy depletion and even oxygen tension and altering gene expression and metabolism in order for the cell to adjust, and one of those key metabolic and gene expression pathways that it regulates is protein synthesis, primarily through its target 4E-BP, one of the earliest known targets shown to be directly phosphorylated by mTOR [9, 75]. In most cells, there exists three 4E-BP isoforms (4E-BP1, 2 and 3) and all are phosphorylated on multiple serine and threonine residues by mTOR. 4E-BPs act as small molecular mimics to eIF4G, bearing the canonical Tyr-X-X-X-X-Leu-ϕ eIF4E binding consensus motif (where ϕ represents a hydrophobic residue) [76]. Under nutrient replete and growth-factor abundant conditions, where mTOR activity is turned on, 4E-BP is hyperphosphorylated and can no longer bind to eIF4E allowing for robust translation initiation. However, upon growth factor withdrawal or nutrient starvation, mTOR activity is reduced, leading to the dephosphorylation of 4E-BP, allowing for tight binding to eIF4E (since they are no longer sterically hindered by the phosphate charges), blocking access to eIF4G and thereby inhibiting translation. Although much of the regulation of protein synthesis derived from signaling through mTOR acts predominantly through the 4E-BPs [77], mTOR can also modulate translation initiation and eIF4F through phosphorylation of p70 S6 kinase (or S6K) and its downstream substrates Pdcd4 [78] and eIF4B [79]. Pdcd4 was first identified as tumor suppressor in the clonally derived JB6 cell line that had lost the ability for neoplastic transformation [80]. It was subsequently found to bind to both eIF4A and eIF4G and thus inhibits eIF4A helicase activity while preventing its association with eIF4G, restricting translation [81]. Following activation of mTOR, Pdcd4 is phosphorylated on Ser67 by S6K1, which in turn promotes phosphorylation of Ser71 and Ser76 allowing for binding to βTRCP (F-box proteins) and ubiquitin-mediated degradation [78]. This releases eIF4A and concomitantly stimulates cap-dependent translation [78]. eIF4B is also a substrate of S6K, whose phosphorylation can also affect translation, but its effects are far from obvious [79, 82].

The question arises as to which of these two interactions (4E:4E-BP and eIF4A:Pdcd4) is rate-limiting (and therefore more important) *in vivo* for eIF4F assembly. The answer may be that it depends on context or that both are important but affect translation initiation in different ways. Comparing relative abundance of factors (eIF4A is the most abundant translation initiation factor present at three copies per ribosome [83] and eIF4E is the least abundant present at 0.26 copies per ribosome [83]) does not address this issue since subcellular localization could render abundant factors rate-limiting. The relative stoichiometry of eIF4E and 4E-BPs versus that of Pdcd4 and eIF4A has not been reported and may differ depending on cell type. Another point to consider is that the cap structure is required to stimulate translation initiation, but its presence is not an absolute requirement for translation initiation *in vitro* or *in vivo* [84-92]. De Gregorio et al. [93] have shown that eIF4G mutants lacking the eIF4E binding site can activate translation on uncapped mRNA reporters *in vitro* and that the central core domain of eIF4G (containing one eIF4A and eIF3 binding site) is sufficient to mediate ribosome recruitment [94]. The 5' end specificity that is observed upon translation of uncapped transcripts may be imparted because the mRNA is masked by RNA binding proteins [89]. These results suggest a model whereby blocking eIF4E from entering into the eIF4F complex could still allow for translation because in principle, eIF4G:eIF4A mediated, 5'-end directed translation initiation could still occur, albeit at a reduced rate. Removal of the eIF4A subunit from the eIF4G:eIF4A dimers or from the eIF4F complex would result in stronger repression or translation inhibition of a different set of mRNA transcripts. Indeed, we propose that differences in translation inhibition observed between cell lines exposed to rapamycin [95-98] in part may be a consequence of which step (4E:4E-BP or eIF4A:Pdcd4) is predominantly affected upon mTOR inhibition.

Clearly, a cancer cell would benefit greatly from hyperactive mTOR activity and this is what is observed in many forms of cancer. In fact, mutations in upstream regulators of mTOR (e.g. PTEN, Akt, PI3K etc.) are some of the most frequently observed (see for example Ref [99]). Thus, cancers driven by hyperactive mTOR signaling would be hypersensitive to treatment with rapamycin, and this is often the case in cell culture [100] and mouse models [98, 101]. In the clinic, however, rapamycin treatment has had much more modest success. Although the rapamycin analog everolimus (RAD001) was recently approved by the FDA for the treatment of advanced stage renal cell carcinoma [102], overall clinical outcome with rapamycin analogs are unpredictable and in general are only marginally effective as monotherapy [103]. The reasons behind rapamycin's struggles in the clinic are not yet clear, but might relate to its ability to reactivate PI3K/Akt signaling upstream of mTOR, negating a well known mTOR negative-feedback loop [104-106]. In addition, recent data has suggested that rapamycin's ability to inhibit mTOR kinase activity (and downstream 4E-BP phosphorylation) is only partial, suggesting that catalytic-site inhibitors might prove more effective [107, 108]. Additionally, eIF4E has been shown to be a genetic modifier of the rapamycin response, with increased levels of eIF4E imparting rapamycin resistance [109]. As well, the status of 4E-BP1 also appears to contribute to mTOR's oncogenic repertoire. Recent publications have suggested that 4E-BP1 is vital for the regulation of cell proliferation by mTOR [77] and 4E-BP1 inactivation contributes to growth in mouse tumor models [33, 77, 110]. Moreover, the expression of a nonphosphorylatable, constitutively active 4E-BP1 suppressed growth of tumors driven by PI3K and K-Ras mutations [111]. Taken together, these studies demonstrate that development of novel compounds and strategies targeting eIF4F-dependent translation directly would be a feasible therapeutic strategy in the treatment of cancer.

### eIF4E Cap-Analog Inhibitors

Oldest among inhibitors of eIF4F are synthetic nucleotide cap-analogs, those that compete for binding with nascent capped transcripts to eIF4E. These have been used extensively now for over 30 years in the field and have been instrumental in the identification of specific translation initiation factors and the elucidation of the process of initiation as a whole [15, 112]. Diverse chemical modification to cap-analogs and nucleosides have given rise to a myriad of novel structures with even greater inhibitory potentials than the common synthetic cap-analog precursor (m^7^GpppG) or even ordinary methyl-7-GTP (m^7^GTP) [113] but their use has been limited to *in vitro* studies since they are not readily cell membrane permeable nor stable in cell culture [114, 115]. In an effort to circumvent such pitfalls, current research has focused on the use “pronucleotide” modifications (nucleosides with protecting groups that convert to their active metabolic form by native cytosolic enzymes) in the development of cap-analogs with greater therapeutic potential. In a recent paper, Ghosh et al. [116] have reported the synthesis of phosphoramidate derivatives of m^7^GTP, a “pronucleotide” modification that is relatively non-toxic, water soluble and much more stable in blood plasma. One compound, dubbed 4Ei-1 (Table I), was reportedly capable of inhibiting cap- and eIF4E-dependent reporter constructs both *in vitro* and *in vivo* when injected into freshly fertilized zebrafish. Although promising, these results are still somewhat incomplete since they have yet to determine whether 4Ei-1 can natively cross phospholipid membranes or inhibit translation in mammalian experimental systems.

**Table I:** Summary of Translation Initiation Inhibitors Targeting eIF4F

Compound	Structure/Sequence	Target	Mode of Action	Reference
Pateamine	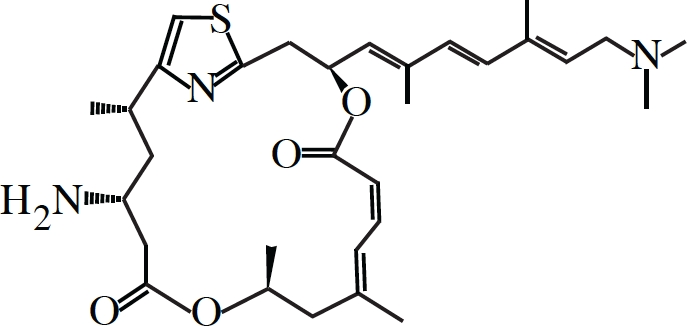	eIF4A	Chemical Inducer of Dimerization (CID)	(126, 129, 130, 131)
Hippuristanol	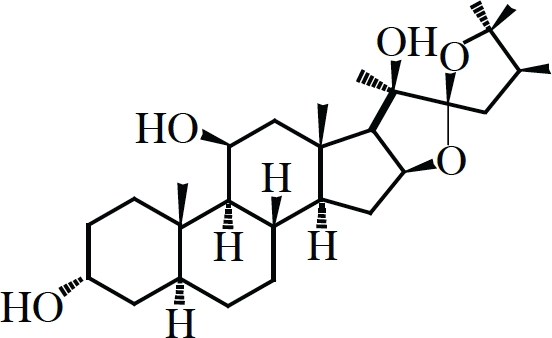	eIF4A	Inhibition of mRNA binding	(127, 132)
Silvestrol	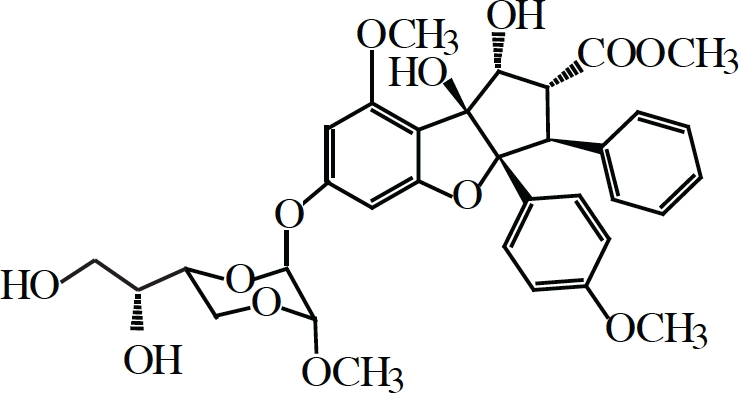	eIF4A	Chemical Inducer of Dimerization (CID)	(128, 133, 134, 135)
4EGI-1	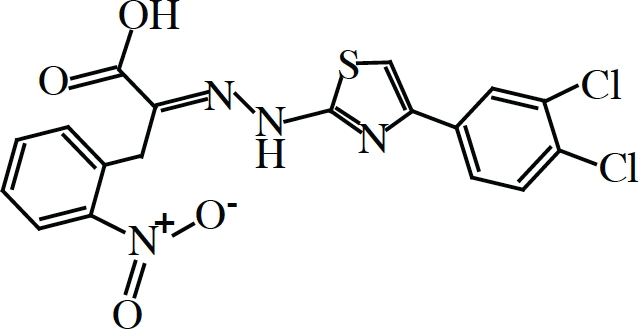	eIF4E	Inhibition of eIF4E:eIF4G interaction Stimulation of eIF4E:4E-BP1 interaction	(122)
4E1RCat	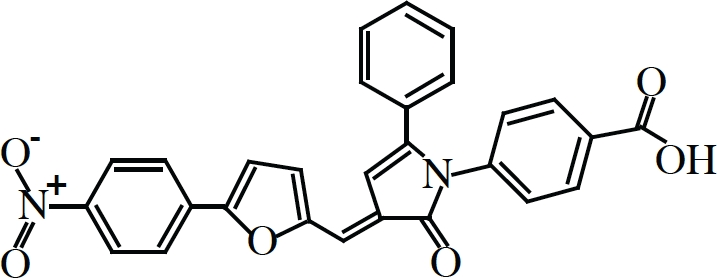	eIF4E	Inhibition of eIF4E:eIF4G interaction Inhibition of eIF4E:4E-BP1 interaction	(123)
4Ei-1	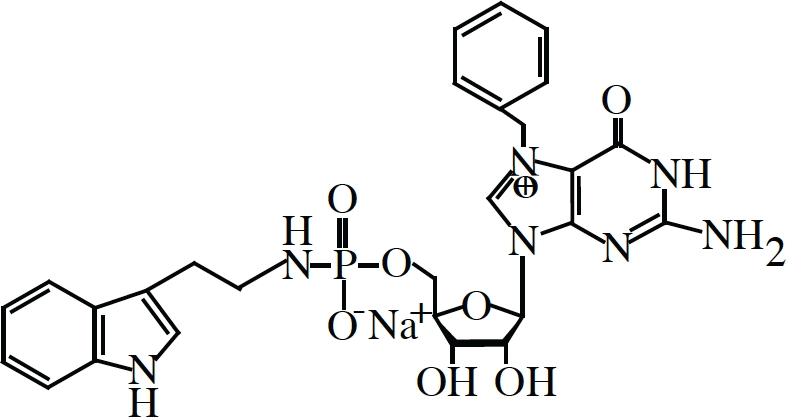	eIF4E	Cap analogue-Competition with 5′ mRNA Cap structure for binding to eIF4E	(116)
4E-ASO4	5′-TGTCATATTCCTGGATCCTT-3′	eIF4E	Antisense oligonucleotide-reduction of protein production	(125)

Another nucleoside analog, ribavirin, has recently garnered some attention as a novel anti-eIF4E cancer therapeutic [117, 118]. Ribavirin is a nucleoside analog that displays fairly broad anti-viral properties and is currently FDA approved in the treatment of RSV and HCV. The exact mechanism of action of ribavirin in the cell has remained somewhat obscure, with theories ranging from ribavirin acting as a direct inhibitor of viral transcription or mRNA capping to a more indirect method of inhibition by reducing intracellular GTP pools or by promoting an interferon response [119]. A paper by Kentsis et al. [117] has shown data demonstrating binding of ribavirin to eIF4E in multiple assays, inhibition by ribavirin of eIF4E-dependent functions *in vivo*, as well being able to slow tumor growth in a xenograft mouse model, suggesting that ribavirin can act like a therapeutically viable cap-analog, although this explanation for its pharmacological action has been somewhat controversial [120, 121]. Still, even though mimicking the cellular mRNA cap as a means of directly preventing eIF4E function may seem to be the most “rational” pharmacological approach, it should be emphasized that cellular physiology surrounding the mRNA cap does not begin nor end with translation. Multiple processes governing gene expression, such as pre-mRNA splicing, nucleo-cytoplasmic transport, or mRNA decay (to name a few) utilize the cap structure as point of regulation. Thus, it should always be kept in mind that cap-analogs might have effects other than inhibiting eIF4E-dependant protein synthesis, and that perhaps other pharmacological strategies might prove more specific in design.

### Targeting the eIF4E:eIF4G Interaction

An alternative approach towards the development of agents that can interfere with eIF4E function would be the discovery and design of small molecule inhibitors that would interfere with eIF4E-eIF4G interaction. As of this writing, there are two small molecules that can compete with and block eIF4G from binding to eIF4E. The first of these to be discovered was from the Wagner lab [122], identified in a screen for small molecules that decreased the specific fluorescence polarization of a labeled peptide fragment encompassing the eIF4G binding domain when titrated with recombinant eIF4E. 4EGI-1 (Table I), as it is called, inhibited cap-dependent translation in *in vitro* translation extracts, depleted known eIF4E regulated proteins *in vivo* and elicited apoptosis in several cancer cell lines. NMR spectra further confirmed direct chemical interactions of 4EGI-1 with eIF4E residues. Curiously, 4EGI-1 did not appear to prevent the translational repressor 4E-BP1 from binding to eIF4E (which shares a similar consensus binding motif), but, counterintuitively, seemed to promote the interaction. Whether or not this “gain-of-function” activity contributes to some of the *in vitro* and *in vivo* effects observed remains to be tested.

A second eIF4E-eIF4G inhibitor, found by our group which we named 4E1RCat (Table I), was identified in an ultra high-throughput screen aimed at finding compounds that directly blocked the very same interaction [123]. Here a fluorescence-based assay was used involving recombinant eIF4E and a peptide fragment of eIF4G encompassing the consensus binding motif. Importantly, 4E1RCat blocked the cap-dependent but not HCV IRES-dependent translation of a bicistronic dual-luciferase reporter mRNA with an IC_50_ of ~25 uM [123]. Like 4EGI-1, 4E1RCat can prevent the association of eIF4G to m^7^GTP-Sepharose bound eIF4E, but unlike 4EGI-1, it also blocked 4E-BP1 binding with nearly the same efficacy. 4E1RCat was also pharmacologically active in cells and in mice, where it decreased the rate of overall protein synthesis by ~30%. More promisingly, 4E1RCat, like other protein synthesis inhibitors (see below), was able to improve the response to chemotherapy in the Eμ-myc mouse lymphoma model and thus prolong the animal's tumor-free survival, without any obvious toxicity.

### Targeting eIF4E Production

An alternative to using competitive small molecule inhibitors would be the use of bioavailable oligonucleotides to genetically suppress the production of eIF4E protein. Earlier work demonstrated the feasibility of using antisense oligonucleotides (ASO) targeting eIF4E, thus limiting the tumorigenicity of K-Ras transformed cell lines, but was constrained technologically to proof-of-principle *in vitro* manipulations [124]. More recently, Graff and colleagues at Eli Lilly Research Labs [125] have developed ASOs directed against eIF4E using second-generation backbone antisense modifications that impart improved nuclease resistance and tissue stability to allow for effective systemic therapeutic delivery. In their study, they observed 80% knockdown of eIF4E with their most potent 4E ASO (Table I), which had only a relatively small impact on global protein synthesis (~20% change). Still, known eIF4E-specific pro-growth and pro-survival gene products decreased in a dose-dependent manner. Impressively, administration of 4E ASOs almost completely blocked tumor growth in breast and prostate xenografts. Furthermore the reduction of eIF4E also appeared to prevent endothelial cell tube formation suggesting a possible role for eIF4E in tumor angiogenesis. And from a pharmacological standpoint, reduced eIF4E levels were well tolerated in normal mouse tissues, perhaps reflecting the notion that the sensitivity and tumor selectivity from the specific translational downregulation of oncogenic targets afforded from eIF4E knockdown allows for a greater therapeutic index.

### Targeting eIF4A activity

Loss of eIF4E prevents binding of eIF4F to the cap-structure that ultimately reduces its ability to eliminate 5'-end RNA secondary structure and hampers efficient recruitment of the 43S ribosome complexes. A different approach towards limiting translation initiation would be by targeting eIF4A, the source of eIF4F enzymatic activity. Our lab has previously reported on the identification and characterization of three small molecule inhibitors of eIF4A (Table I) [126-128]. Two of these (pateamine A and silvestrol), paradoxically, stimulate eIF4A activity, mostly by forcing the binding of eIF4A to RNA on which much of its enzymatic activity relies [126, 128-131]. The net effect is however an overall reduction in protein synthesis caused by a pronounced block in translation initiation as a result of most of eIF4A being depleted from the eIF4F complex [126, 128, 129]. Hippuristanol, the third eIF4A inhibitor, suppresses eIF4A's helicase, ATPase and RNA binding properties[127, 132]. All three compounds show high specificity towards eIF4A, making them attractive for studying the biological action of eIF4A *in vivo*.

Silvestrol has been tested in several different mouse cancer models with encouraging results. The compound does not cause distress, weight loss or liver damage, and does not appear to immunosuppress in the mouse [133, 134]. In some settings, silvestrol alone has therapeutic benefit as chemotherapy: this is the case for xenograft studies of acute lymphoblastic leukemia in SCID mice [134], prostate cancer and breast cancer xenografts in nude mice [133], in the Eμ-Tcl-1, a mouse model of chronic lymphocytic leukemia (CLL), as well as primary human CLL samples [134]. In the latter study, the authors observed that silvestrol was far more toxic towards B-cells than T-cells and that B-cells derived from chronic lymphocytic leukemia patients were more sensitive to the drug than from healthy individuals [134], suggesting preferential targeting of faster growing leukemic cells by silvestrol. Still, in the Eμ-Myc mouse model, a Burkitt's B-cell lymphoma cancer model, silvestrol alone showed no effect, although this might be explained by the lower doses used [128]. However, when combined with doxorubicin it greatly prolonged the tumor-free survival of tumor-burdened mice [128]. Moreover, this combination therapy was even effective against eIF4E-driven lymphomas, which normally do not respond to conventional monotherapy or combination chemotherapy regime [109]. This is also what is seen in AML cells, where silvestrol increases the cytotoxicity of daunorubicin, etoposide and cytarabine [135].

## FUTURE DIRECTIONS

Even though these are still very early days in the development of eIF4F inhibitors as antineoplastics, protein synthesis inhibitors seem poised for a pharmaceutical comeback. Homoharringtonine (HHT) (omacetaxine mepesuccinat), a known inhibitor of peptide-chain elongation, has shown promise in Phase II clinical trials for the treatment of gleevec-resistant chronic myelogenous leukemia (CML) [136] and was approved to be “fast-tracked” by the FDA for clinical development. Homoharringtonine's anti-neoplastic abilities have been known since the ‘70s [137] and it was considered the premier drug in salvage therapy for CML before getting supplanted by gleevec [138]. Recent data have suggested that part of HHT's mechanism of action is its ability to downregulate the short-lived anti-apoptotic and pro-oncogenic protein Mcl-1 [139, 140], as would be expected from transient exposure to a general protein synthesis inhibitor and perhaps explaining some of the synergistic effects observed from most inhibitors of translation elongation in the Eμ-Myc model [141]. Other elongation inhibitors have had less success clinically in treatment of cancer due to non-specific toxicity in the patient [142], suggesting a limited therapeutic index for these general protein synthesis inhibitors. Targeting eIF4F might have the benefit of expanding this window of therapeutic success, given the molecular response afforded by blocking translation initiation: one can affect the scope of protein synthesis production (from a more global to more specific mRNA translational inhibition) not only from dosing of a given drug but also by changing the nature of the pharmacological intervention (eIF4A versus eIF4E inhibition). It will be interesting to see whether targeting other regulators of initiation (e.g.-eIF4B. eIF4H, PABP) would yield a similar or even greater therapeutic potential in the treatment of cancer. The next 5 years will indeed be exciting as we monitor the clinical development and progress of translation initiation inhibitors as potential antineoplastics.
